# Breast Implant-associated Anaplastic Large Cell Lymphoma: A Review with Emphasis on the Role of Brentuximab Vedotin

**DOI:** 10.33696/immunology.2.025

**Published:** 2020-05

**Authors:** Anthony Stack, Nadia Ali, Nadia Khan

**Affiliations:** 1 Department of Internal Medicine, Temple University Hospital, Philadelphia, PA USA; 2 Department of Medicine, section of Hematology, Temple University Hospital, Philadelphia, PA, USA; 3 Department of Hematology and Oncology, Fox Chase Cancer Center, Philadelphia, PA USA

**Keywords:** Lymphoma, ALCL, Brentuximab vedotin, Breast Implant associated Anaplastic Large Cell Lymphoma, BIA-ALCL

## Abstract

Breast implant-associated anaplastic large cell lymphoma is a recently recognized complication of textured breast implants. It typically presents as unilateral peri-implant swelling approximately 7-10 years after implantation. While the course is usually indolent, breast implant-associated anaplastic large cell lymphoma may form a locally invasive mass and metastasize to regional lymph nodes or beyond to distant sites. Surgical excision has been well established as the standard of care for localized disease; however, guidelines directing management of advanced, recurrent or unresectable disease are based on limited and extrapolated evidence. The CD30-targeting immunoconjugate, brentuximab vedotin, has been utilized in this setting, typically in combination with chemotherapy. We recently reported a patient with unresectable breast implant-associated anaplastic large cell lymphoma who was treated with brentuximab vedotin monotherapy and has now sustained complete remission for 2.6 years. Herein, we provide an up-to-date review of the epidemiology, pathogenesis, clinical features, diagnosis and management of breast implant associated anaplastic large cell lymphoma with emphasis on the role of brentuximab vedotin.

## Introduction

Anaplastic large cell lymphoma (ALCL) represents a heterogeneous group of T-cell lymphomas, which characteristically express CD30 and are associated with translocations involving the *anaplastic lymphoma kinase (ALK)* gene on chromosome 2p23 [1]. Systemic ALCL, which may be subclassified by the presence or absence of ALK gene expression, and the more indolent primary cutaneous ALCL, characterized by ALK negativity, have been well described. In 2016, the World Health Organization expanded the definition of ALCL to provisionally include breast implant-associated anaplastic large cell lymphoma (BIA-ALCL) as a distinct subclassification, in recognition of its distinct etiology and clinical course [2].

The index case of ALCL associated with a breast implant was reported in 1997 by Keech and Creech, who described a woman presenting with a mass in her right breast four years after breast implantation [3]. In the years that followed, numerous additional cases of ALK negative ALCL were reported in association with breast implants. All forms of ALCL are rare, with an estimated annual incidence of 1 in 500,000; thus, the apparent preponderance of cases associated with textured breast implants prompted further investigation into a possible causal relationship [4]. In light of mounting data supporting this association, the Food and Drug Administration (FDA) released a safety communication in 2011, summarizing the scientific data regarding the association between breast implants and ALCL and advising provider and patient vigilance until this association could be better characterized [5]. In August of 2012, the American Society of Plastic Surgeons (ASPS), The Plastic Surgery Foundation (PSF) and the FDA collaborated to create the “*Patient Registry and Outcomes For breast Implants and anaplastic large cell Lymphoma (ALCL) etiology and Epidemiology*” (PROFILE) registry, in order to collect demographic, clinical and follow up data on cases of BIA-ALCL within the United States.

Epidemiologic data from the PROFILE registry and other data collecting agencies worldwide has suggested a strong association between textured breast implants and the development of BIA-ALCL, garnering significant scientific, political and media attention [6-8]. A growing concern for the safety of textured breast implants has led a number of countries, including France, Canada, Australia and Egypt to restrict the sale of textured implants over the last two years. On July 24th, 2019, the United States FDA requested a voluntary recall of certain textured implants due to an elevated risk for BIA-ALCL. The first world consensus conference on BIA-ALCL, held on October 6, 2019 in Rome, Italy, provided an up-to-date report of the known literature from many of the worlds experts and additional commentary on the need for global industry review.

As recognition of BIA-ALCL has expanded, there has been a growing need for guidelines to direct diagnosis and treatment. In 2016, the National Comprehensive Cancer Network (NCCN) published consensus guidelines for the diagnosis and treatment of BIA-ALCL, based on limited experience from large centers such as the MD Anderson Cancer Center [9]. In 2019, these guidelines were updated to reflect an exponential expansion of the literature on the natural history, diagnosis and management of BIA-ALCL [10].

It has been well established that surgery provides the greatest benefit in terms of both survival and prevention of recurrence; however, there has been little guidance available for clinicians faced with cases of advanced, unresectable BIA-ALCL [11]. Interest has been given to the utilization of the novel anti-CD30 antibody-drug conjugate, brentuximab vedotin; however, until recently, its use has only been described in case reports and small series as an adjuvant for limited stage disease or as combination therapy with conventional chemotherapy for advanced disease [12-14]. Recently, we published a case report describing the successful application of brentuximab vedotin as monotherapy after surgical explantation and radiation for a case of advanced, unresectable BIA-ALCL [15]. In the current review, we will provide and in-depth discussion of the current literature on BIA-ALCL and the evidence supporting the use of brentuximab vedotin in treatment.

## Epidemiology

Multiple epidemiologic studies have implicated implant texturing as complicit in the formation of BIA-ALCL. Texturing of implants began in 1987, in an effort to improve contracture resistance and increase the rotational stability of implants in situ [16]. While many published reports do not contain information on the type of implant utilized, multiple studies have shown that all patients for whom this data was available had a history of at least one textured implant prior to diagnosis [16-18]. Furthermore, risk seems to be correlated to implant surface area and roughness, with implants utilizing polyurethane foam and salt-loss texturing contributing the highest risk [19].

Several factors have made it difficult to accurately estimate the risk for BIA-ALCL, including difficulty in determining the prevalence of breast implants, variability in reporting and awareness of BIA-ALCL among clinicians and the delay in onset between implantation and disease presentation [20]. Between September, 2018 and July, 2019, there was a 25% increase in the number of Medical Device Reports of BIA-ALCL reported to the FDA, totaling 573 unique cases, worldwide [21]. In the largest prospective series of textured implants to date, including 17,656 patients, eight patients were found to develop BIA-ALCL, a risk of 1 in 2,207 (95% CI 1,120 to 5,112) [22,23]. Another retrospective study of 100 confirmed cases in the US as of 2015 estimated a lifetime prevalence of 33 per million (~1 case per 30,000) women with textured breast implants [24]. A Danish study estimated a relative risk of BIA-ALCL in women with breast implants at 421.8 and a lifetime risk of 1 per 35,000 women with implants at age 50 and 1 per 12,000 at age 70 [25]. Notably, the latter of these studies did not distinguish textured from smooth implants and therefore may underestimate risk by increasing the denominator.

Average age of onset has been consistent among various studies at around 50 years, with the time from device implantation to disease presentation typically ranging from around 7 to 10 years; however, cases have been reported as early as 4 months after implantation [6,11,16,17,19,24]. Reason for implantation (reconstructive vs. cosmetic), type of implant fill (silicone vs. saline) or implant size does not seem to influence risk [5,26]. A racial/ethnic predisposition has been proposed, with much lower incidence having been reported among those of Asian, African and Native American descent [20,27].

## Pathogenesis

**Figure 1:**
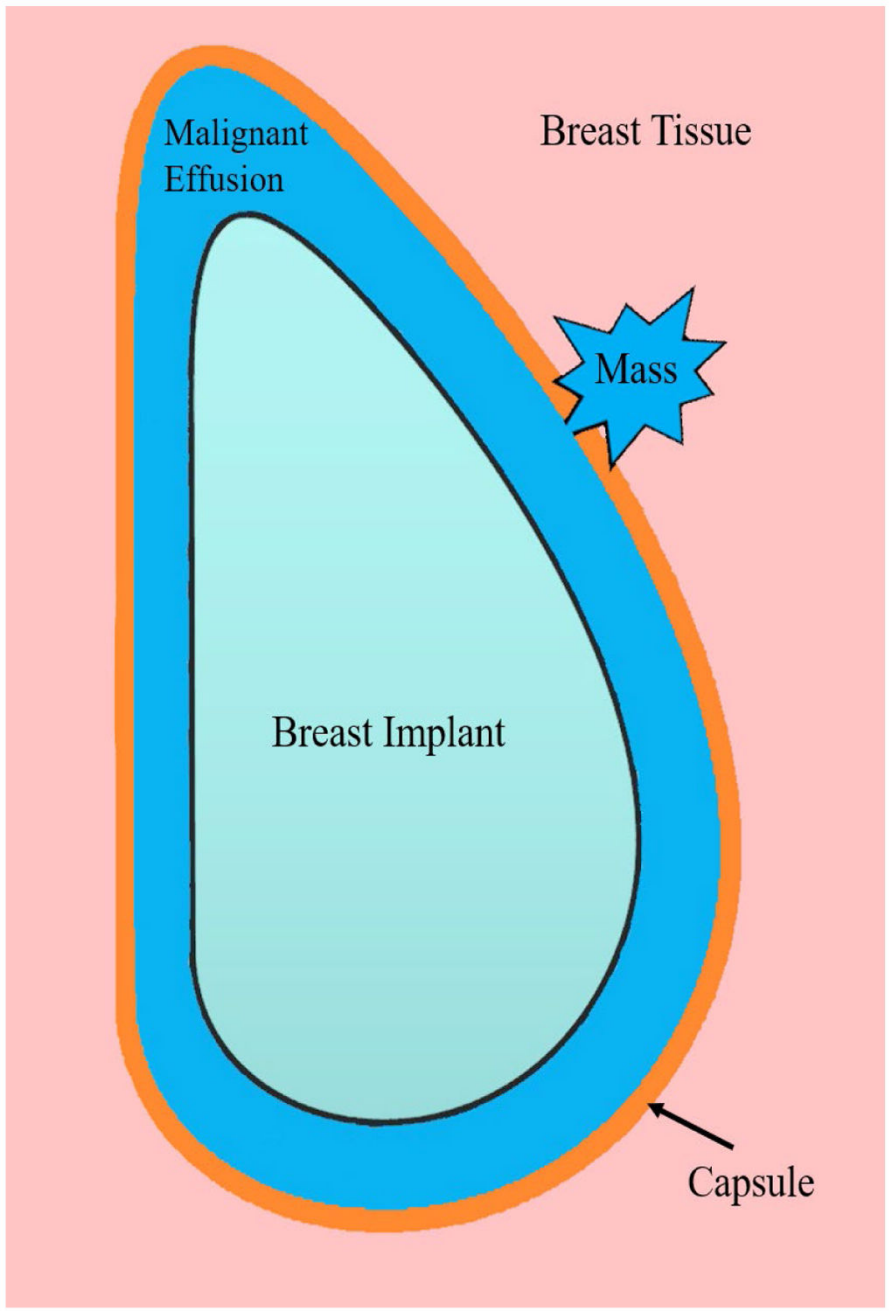
Proximity of BIA-ALCL to surrounding structures. After breast implantation, a fibrous capsule forms around the breast implant as part of a normal inflammatory foreign body response. BIA-ALCL typically forms as a malignant effusion between the breast implant and surrounding capsule. With progression, malignant cells coalesce into a mass, which may invade the capsule into surrounding tissues.

The pathological events which give rise to BIA-ALCL are thought to stem from chronic inflammation. Upon implantation, a fibrous capsule is formed around the breast implant material as part a primarily T-cell driven inflammatory response [28]. While this process does not itself represent a pathological event, this is thought to create the substrate on which certain inflammatory drivers may act to promote malignant transformation (Figure 1). In keeping with this theory, CD30+ clonal T-cells have been identified within the capsule of a benign late-seroma, suggesting a progressive pathway from benign lymphoproliferative disorder to BIA-ALCL [29].

Since BIA-ALCL has been shown to occur only in patients with textured implants; studies have attempted to define the link between implant texturing and inflammation. One theory is that bacterial contamination may contribute to lymphomagenesis. In 2015, Hu et al. demonstrated a significantly greater number of bacteria (via polymerase chain reaction identification of total bacterial 16S RNA) on textured versus smooth breast implants after porcine implantation [30]. Furthermore, the authors demonstrated significantly more T-lymphocytes on textured implants, with the number of lymphocytes being linearly correlated with the bacterial load. CD4^+^ T-cells taking on a Th17/Th1-like phenotype, with dual expression of IFN-γ and IL-17F, have been suggested as the origin of BIA-ALCL [16,31]. Interestingly, CD4^+^ T-cells showed the strongest correlation with the number of bacteria. A subsequent study has suggested that the species of bacteria predominating on implant biofilms may play a role in stimulating the disease. The gram-negative bacillus, Ralstonia, has been found to predominate on BIA-ALCL capsular specimens, compared with primarily staphylococcal species found on non-malignant capsules [32]. While this data was initially promising, a 2019 study which compared the microbiome of 7 BIA-ALCL patients with both contralateral and non-lymphoma controls found no difference in the microbiomes of BIA-ALCL implants, arguing against the theory that certain bacterial species predispose to BIA-ALCL [33].

Others have suggested that genetic predisposition may underlie some cases of BIA-ALCL. Somatic mutations of the JAK/STAT3 pathway have been found to be major drivers of many tumors derived from inflammatory conditions, as well as in systemic and cutaneous forms of ALCL [34,35]. STAT3 phosphorylation, which occurs either directly through IL-6 receptor activation or indirectly via infectious or other inflammatory mediators, promotes T-cell transition to the TH17 phenotype [34]. It also acts to increase transcription of multiple genes involved in tumorigenesis, including those involved in apoptosis prevention, proliferation, angiogenesis and metastasis. While the chimeric ALK protein has been shown to activate the STAT3 pathway directly, ALK-negative forms of ALCL have been shown to commonly activate this pathway via mutations in JAK1, STAT3 and fusion proteins involving TYK2 and ROS1 [35]. Indeed, multiple series have shown phosphorylated (activated) STAT3 is characteristically present in BIA-ALCL, while typical ALCL rearrangements (ALK, DUSP22 and TP63) are characteristically absent [36,37]. In keeping with this theory, Blombery et al. recently demonstrated activating somatic mutations in JAK1 and STAT3 in 2 cases of BIA-ALCL through whole exome sequencing, as well as a germline JAK3 variant in one case, suggesting a possible congenital predisposition [38].

Notably, the pathological events leading to BIA-ALCL may not be unique to breast implants. Similar phenomena have occurred in the setting of other inflammatory seromas, suggesting a parallel etiology. A recent case of gluteal implant-associated, ALK negative ALCL has been described, occurring 1 year after placement of textured, silicon gluteal implants [39]. Similarly, another case of effusion-associated ALCL occurred in a woman without breast implants, who developed an ALK-negative ALCL in the background of an aspirated benign breast cyst [40]. These cases highlight the gaps in our current understanding of the T-cell malignant transformation process and provide further support for the study of BIA-ALCL as a model for this phenomenon.

## Clinical Presentation, Diagnosis and Staging

The most common presentation of BIA-ALCL is as a localized late peri-implant effusion, which occurs in approximately two thirds of patients [41]. This typically manifests clinically as unilateral breast asymmetry and discomfort approximately 7 to 10 years after implantation, although cases occurring at intervals as short as one to four months after repeat implantation have been reported [6,11,16,18]. Less commonly, patients may present with a discrete mass (typically indicating tumor extension beyond the capsule), regional lymphadenopathy [17], breast ulceration [16], skin papules [42] or serendipitously during revision [6]. Systemic symptoms, such as fevers, weight loss or night-sweats are rare but have been reported [6]. A recent study of 70 BIA-ALCL patients reported a 20% rate of lymph node involvement, most often of the axillary chain, portending a worse prognosis [43].

Recently, guidelines have been established by the NCCN for the diagnosis and management of BIA-ALCL. Patients presenting with late onset peri-implant seromas, masses or ulceration (> 1 year after implantation) should undergo further testing, as prospective studies have suggested that 9% of these patients will have an underlying BIA-ALCL [9,44]. Initial workup should include breast ultrasound, with MRI in selected cases. Adrada et al. investigated several imaging modalities in the diagnosis of BIA-ALCL and found that ultrasound and MRI show similar sensitivities for detecting effusions (84% vs 82%, respectively) and masses (46% vs. 50%, respectively), while CT and mammography fell short of other modalities [45]. In our case, ultrasound failed to demonstrate a mass and it was only after confirmation with MRI that this was discovered, suggesting the utility of a sequential hierarchy of imaging based on clinical suspicion.

While laboratory testing currently plays little role in the diagnosis of BIA-ALCL, researchers have been attempting to find a sensitive and specific screening test for patients presenting with late seromas. Recently, Hanson et al. showed that a novel rapid enzyme-linked immunosorbent assay (ELISA) screening test on seroma fluid was shown to be 100% sensitive and specific among 9 pathologically confirmed patients with BIA-ALCL and 7 controls [46]. Interestingly, they also demonstrated circulating CD30 in undiluted plasma. Detection of circulating CD30 has already been demonstrated as a marker for certain autoimmune and parasitic infections and, if proven viable for BIA-ALCL, would provide a noninvasive method for early detection of these patients in resource limited settings [47]. Notably, CD30^+^ T-cells have been detected in seroma fluid and serum of patients with benign late seromas, possibly complicating the use of CD30 as a specific disease marker [29].

Confirmed effusions or masses should undergo tissue sampling with fine-needle aspiration or biopsy, respectively. Diagnosis requires demonstration of T-cell clonality, confluent CD30 positivity and characteristic cellular morphology [8]. Histologically, cells of BIA-ALCL are similar to those of systemic ALCL, characterized by pleomorphic and anaplastic morphology and eosinophilic cytoplasm [48]. They are further characterized by negativity for the ALK protein or translocation involving the *ALK* gene [49].

Upon diagnosis, preoperative positron emission tomography computed tomography (PET/CT) is recommended for staging and surgical planning. Like most lymphomas, BIA-ALCL was initially staged using the Ann Arbor system; however, this has proven to be less applicable given the clinical behavior of BIA-ALCL. For instance, BIA-ALCL rarely disseminates, but rather forms a mass and invades local tissues and lymph nodes; more analogous to a solid tumor than a lymphoma. In fact, Clemens et al. found that more than 80% of their cohort of 87 BIA-ALCL patients would be classified as Ann Arbor stage I, limiting this systems utility to predict prognosis and direct treatment [11]. For this reason, many more recent reports have utilized their alternative staging criteria, modeled after the American Joint Committee’s TNM staging system for solid tumors (Table 1). Using this system, the rate of events (lymphoma persistence, recurrence, progression, relapse or patient death after appropriate treatment) and overall survival was better predicted by the TNM staging system, when compared to the Ann Arber system [11]. This TNM staging classification is now included in the 2019 update of the NCCN guidelines. By these criteria, our patient would have been stage IIE-bulky by the Ann arbor system and stage III via the TNM system [50].

## Management

The initial approach to BIA-ALCL is similar to the management of solid malignancies. Since patients who are able to undergo complete resection have a better prognosis, the most important first step in management is to determine whether the disease can be completely resected [11,51]. A multidisciplinary team approach should be used whenever possible [5]. Complete surgical excision has been retrospectively shown to significantly prolong both overall survival (OS) and event free survival compared to other interventions and is considered the standard of care for localized BIA-ALCL [11].

Patients with residual disease after surgery should be offered adjuvant radiation with or without systemic therapy [10]. Systemic therapy approaches have been extrapolated from treatment for systemic ALCL and typically include an anthracycline based regimen. Recently, the NCCN has suggested adjuvant regimens include brentuximab vedotin, which has demonstrated efficacy for BIA-ALCL in case reports [12-15]. Further evidence for the use of brentuximab vedotin is extrapolated from the ECHELON II trial, which demonstrated an OS benefit when brentuximab vedotin was added to anthracycline based chemotherapy versus chemotherapy alone for CD30+ peripheral T-cell lymphomas [52]. Based on the results of this trial, the NCCN preferred treatment regimen for systemic ALCL is chemoimmunotherapy with brentuximab vedotin and cyclophosphamide, doxorubicin, and prednisone (CHP). CHOP, CHOEP and dose adjusted EPOCH are other recommended regimens.

## Brentuximab Vedotin

**Figure 2:**
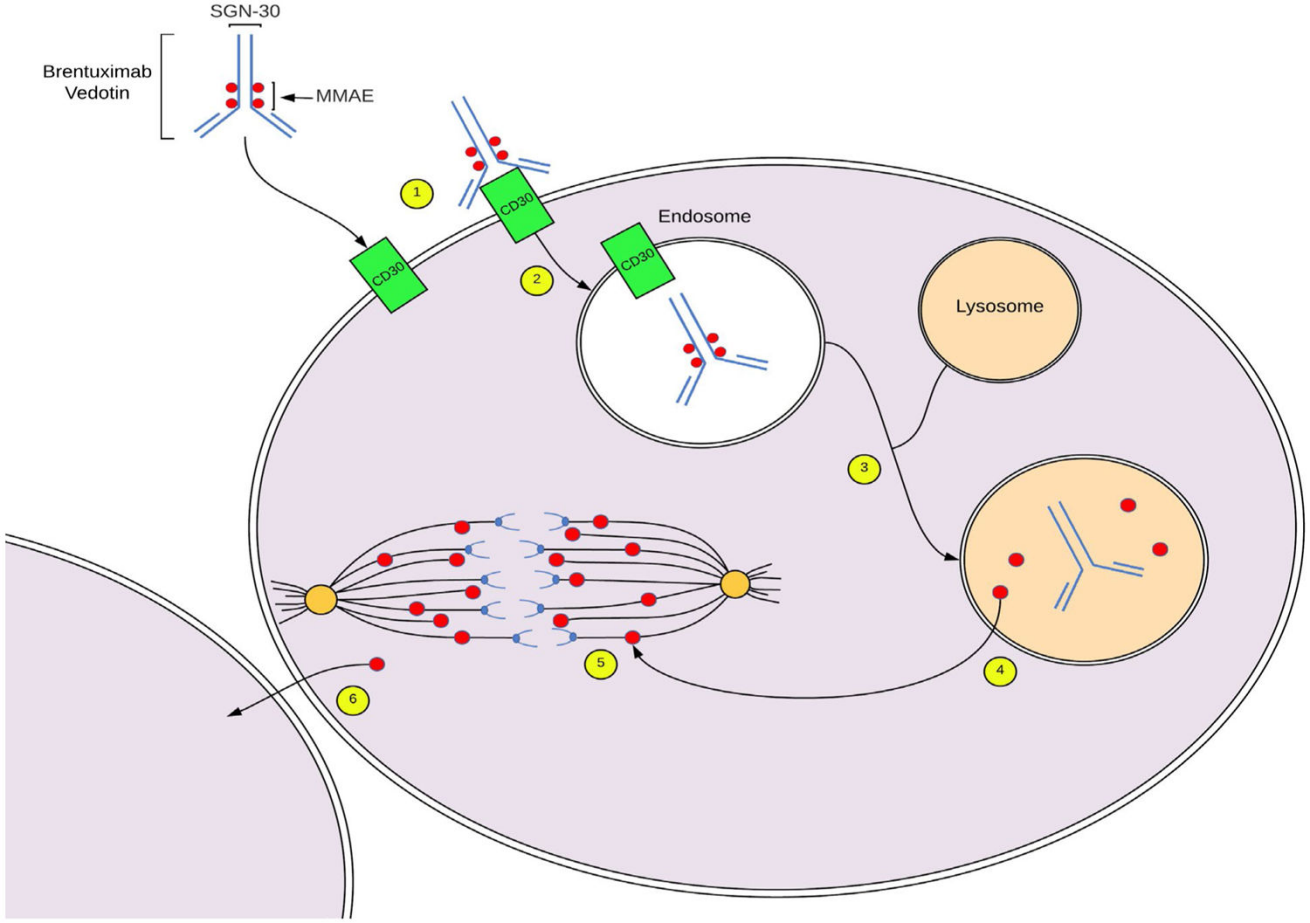
Mechanism of action of brentuximab vedotin. **1.** Brentuximab vedotin binds CD30 on lymphoma cell membranes. **2.** The antibody-receptor complex is internalized via clathrin-mediated endocytosis, forming an endosome. **3.** The endosome undergoes lysosomal fusion. **4.** Within the endolysosome, MMAE undergoes enzymatic cleavage by cathepsin. **5.** MMAE inhibits microtubules by disrupting tubulin polymerization, causing G2/M cell cycle arrest and subsequent apoptosis. **6.** Some MMAE may diffuse into neighboring cells, magnifying its antineoplastic effects. MMAE, Monomethyl Auristatin E.

Brentuximab vedotin is a CD30 targeting immunoconjugate which delivers the anti-tubulin agent, monomethyl auristatin E (MMAE) to targeted cells [53]. Its target, CD30, is a type I transmembrane receptor protein whose expression is characteristic in all types of anaplastic large cell lymphoma but whose expression in benign tissues is limited to activated and virally infected lymphocytes and certain cells of the thymic medulla [54]. The function of CD30 in normal cells is poorly understood, as no human disease has been associated with defects in either CD30 or its native ligand, CD153 [54]. The relative preponderance of this antigen on neoplastic cells and rarity of expression in healthy cells make CD30 an ideal target for immunotherapy. Upon binding of brentuximab vedotin to CD30, the receptor-antibody complex undergoes clathrin mediated endocytosis and lysosomal fusion (Figure 2) [55]. Within the lysosome, MMAE is released by proteolytic cleavage and acts to inhibit the assembly and polymerization of microtubules, causing G2/M cell cycle arrest and subsequent apoptosis [54]. Some MMAE may then diffuse into the tumor microenvironment to further act on neighboring cells.

In the United States, brentuximab vedotin is currently approved for previously untreated stage III/IV classical Hodgkin Lymphoma (HL), consolidation therapy after autologous hematopoietic stem cell transplantation for classical HL and relapsed primary cutaneous ALCL or CD30 expressing mycosis fungoides [56]. Recently, brentuximab vedotin was also approved for frontline treatment of systemic ALCL and other CD30-expressing peripheral T-cell lymphomas after showing an OS benefit when combined with chemotherapy over chemotherapy alone for peripheral T-cell lymphomas [52,57].

Case reports suggest that brentuximab vedotin may also be effective as frontline monotherapy, either adjuvantly after surgical excision or as primary treatment for unresectable BIA-ALCL. The safety and efficacy of brentuximab vedotin monotherapy has previously been demonstrated for relapsed and refractory systemic ALCL in a phase II trial [58]. Alderuccio et al. described a patient with stage IIB BIA-ALCL, without capsular invasion, who was treated with surgical excision and adjuvant frontline brentuximab vedotin monotherapy [12]. Their patient remained in complete remission after 3 year follow up. Subsequently, we reported a patient with significant extracapsular invasion, involving both the chest wall and pleura (Stage III) [15]. Our patient received 18 cycles of brentuximab vedotin monotherapy after surgical explantation and radiation therapy. Though her treatment course was complicated by septic shock after the first cycle and peripheral neuropathy after the ninth cycle, she achieved a complete metabolic response which has remained durable for 2.6 years. Given the unique clinical course of BIA-ALCL, more data is required to determine whether brentuximab vedotin monotherapy may take the place of immunochemotherapy for unresectable or refractory BIA-ALCL and clinicians should be encouraged to report their experiences to the PROFILE registry.

## Prognosis

Unlike systemic ALCL, BIA-ALCL tends to follow an indolent course; with an overall 5 year survival rate of 89-92%, compared to 37-49% and 70-93% in ALK− and ALK+ systemic ALCL, respectively [11,17,59]. Patients with a mass, indicating local tumor extension beyond the implant capsule, typically have a worse prognosis. While studies differ in their estimates of prognosis for patients presenting with invasive disease (e.g. mass, tumor positive lymphadenopathy, disseminated or bilateral disease); a cohort of 60 patients showed a 3 and 5 year OS of 82% and 75%, respectively, for patients with a mass, compared to 100% 3 and 5 year OS for patients presenting with capsule-confined disease [17].

## Conclusion

BIA-ALCL is an uncommon and increasingly recognized complication of breast implantation. To date, it has only been reported in association with textured breast implants, prompting international regulation of the breast implant industry. Current treatment guidelines recommend complete surgical excision whenever possible, based on evidence showing that this provides a benefit in terms of both overall and event free survival; however, guidelines directing management of advanced, recurrent or unresectable disease are based on limited and extrapolated evidence. Brentuximab vedotin has shown efficacy for peripheral T-cell lymphomas when combined with chemotherapy and this combination is currently recommended for most patients requiring systemic therapy for BIA-ALCL. Case reports have reported efficacy of brentuximab vedotin as monotherapy for BIA-ALCL in patients unable to receive anthracycline based chemotherapy; however, data supporting brentuximab vedotin monotherapy is lacking. Clinicians should be encouraged to report their experience with brentuximab vedotin for BIA-ALCL to repositories such as the PROFILE registry to allow for refinement of evidencebased guidelines for this uncommon disease.

## Figures and Tables

**Table 1:** TMN staging for Breast Implant-Associated Anaplastic Large Cell Lymphoma [9,11].

TNM Designation and Stage	Description
**T: Tumor Extent**	
T_1_	Confined to effusion or layer on the luminal side of the capsule
T_2_	Early capsule infiltration
T_3_	Cell aggregates or sheets infiltrating the capsule
T_4_	Lymphoma infiltrates beyond the capsule
**N: Lymph Nodes**	
N_0_	No lymph node involvement
N_1_	One regional lymph node involved
N_2_	Multiple regional lymph nodes involved
**M: Metastasis**	
M_0_	No distant spread
M_1_	Spread to other organs/distant sites
**Stage**	
IA	T_1_ N_0_ M_0_
IB	T_2_ N_0_ M_0_
IC	T_3_ N_0_ M_0_
IIA	T_4_ N_0_ M_0_
IIB	T_1-3_ N_1_ M_0_
III	T_4_ N_1-2_ M_0_
IV	Tany Nany M_1_
